# COVID-19 Trends Among Persons Aged 0–24 Years — United States, March 1–December 12, 2020

**DOI:** 10.15585/mmwr.mm7003e1

**Published:** 2021-01-22

**Authors:** Eva Leidman, Lindsey M. Duca, John D. Omura, Krista Proia, James W. Stephens, Erin K. Sauber-Schatz

**Affiliations:** 1 CDC COVID-19 Emergency Response Team.

Coronavirus disease 2019 (COVID-19) case and electronic laboratory data reported to CDC were analyzed to describe demographic characteristics, underlying health conditions, and clinical outcomes, as well as trends in laboratory-confirmed COVID-19 incidence and testing volume among U.S. children, adolescents, and young adults (persons aged 0–24 years). This analysis provides a critical update and expansion of previously published data, to include trends after fall school reopenings, and adds preschool-aged children (0–4 years) and college-aged young adults (18–24 years) (*1*). Among children, adolescents, and young adults, weekly incidence (cases per 100,000 persons) increased with age and was highest during the final week of the review period (the week of December 6) among all age groups. Time trends in weekly reported incidence for children and adolescents aged 0–17 years tracked consistently with trends observed among adults since June, with both incidence and positive test results tending to increase since September after summer declines. Reported incidence and positive test results among children aged 0–10 years were consistently lower than those in older age groups. To reduce community transmission, which will support schools in operating more safely for in-person learning, communities and schools should fully implement and strictly adhere to recommended mitigation strategies, especially universal and proper masking, to reduce COVID-19 incidence.

Children, adolescents, and young adults were stratified into five age groups: 0–4, 5–10, 11–13, 14–17, and 18–24 years to align with educational groupings (i.e., pre-, elementary, middle, and high schools, and institutions of higher education), and trends in these groups were compared with those in adults aged ≥25 years. Confirmed COVID-19 cases, defined as positive real-time reverse transcription–polymerase chain reaction (RT-PCR) test results for SARS-CoV-2, the virus that causes COVID-19, were identified from individual-level case reports submitted by state and territorial health departments during March 1–December 12, 2020.* COVID-19 case data for all confirmed cases were analyzed to examine demographic characteristics, underlying health conditions,^†^ and outcomes. Trends in COVID-19 incidence were analyzed using a daily 7-day moving average, aggregated by week,^§^ and expressed as cases per 100,000 persons.^¶^

Trends in laboratory testing volume and percentage of positive test results were assessed using COVID-19 electronic laboratory reporting data. SARS-CoV-2 RT-PCR test results for May 31–December 12, 2020 were obtained from electronic laboratory reporting data submitted to CDC by health departments from 44 states, the District of Columbia, two territories, and one freely associated state; when information was unavailable in state-submitted data, records submitted directly by public health, commercial, and reference laboratories were used.** Data represent test results, not number of persons receiving tests; test result date was used for analyses. The weekly percentage of positive SARS-CoV-2 RT-PCR test results was calculated as the number of positive test results divided by the sum of positive and negative test results. Because some data elements are incomplete for more than 47% of cases, percentages were calculated only from among those with available information. This project was deemed nonresearch public health practice by the CDC and conducted consistent with applicable federal law and CDC policy.^††^ Analyses were conducted using R software (version 4.0.2; The R Foundation).

During March 1–December 12, 2020, a total of 2,871,828 laboratory-confirmed cases of COVID-19 in children, adolescents, and young adults aged 0–24 years were reported in the United States. Among these cases, the majority (57.4%) occurred among young adults aged 18–24 years; children and adolescents aged 14–17 years accounted for 16.3% of cases, those 11–13 years for 7.9%, those 5–10 years for 10.9%, and those 0–4 years for 7.4% (Table). Overall, 51.8% of cases occurred in females. Among the 1,504,165 (52.4%) children, adolescents, and young adults with COVID-19 with complete information on race/ethnicity, 50.2% were non-Hispanic White, 27.4% were Hispanic/Latino (Hispanic), and 11.7% were non-Hispanic Black. The proportion of cases among Hispanic persons decreased with increasing age from 34.4% among those aged 0–4 years to 24.6% among those aged 18–24 years.^§§^

**TABLE:** Demographic characteristics and underlying conditions among persons aged 0–24 years with positive test results for SARS-CoV-2 — United States, March 1–December 12, 2020

Characteristic	Age group, yrs, no. (%)						
	0–24	0–17	0–4	5–10	11–13	14–17	18–24
**Total**	2,871,828 (100)	1,222,023 (42.6)	212,879 (7.4)	313,913 (10.9)	227,238 (7.9)	467,993 (16.3)	1,649,805 (57.4)
**Sex**							
Female	1,469,744 (51.8)	603,948 (50.0)	100,935 (48.2)	152,494 (49.1)	111,683 (49.7)	238,836 (51.6)	865,796 (53.1)
Male	1,367,271 (48.2)	603,029 (50.0)	108,457 (51.8)	157,769 (50.8)	112,930 (50.3)	223,873 (48.4)	764,242 (46.9)
Other	53 (<0.1)	18 (<0.1)	2 (<0.1)	3 (<0.1)	2 (<0.1)	11 (<0.1)	35 (<0.1)
Missing/Unknown	34,760 (N/A)	15,028 (N/A)	3,485 (N/A)	3,647 (N/A)	2,623 (N/A)	5,273 (N/A)	19,732 (N/A)
**Median age (years)**	19	9	2	8	12	16	21
**Symptom Status**							
Yes	1,247,552 (94.1)	524,390 (91.9)	87,646 (90.4)	126,010 (88.9)	97,831 (91.8)	212,903 (94.5)	723,162 (95.8)
No	77,899 (5.9)	46,166 (8.1)	9,281 (9.6)	15,720 (11.1)	8,736 (8.2)	12,429 (5.5)	31,733 (4.2)
Missing/Unknown*	1,546,377 (N/A)	651,467 (N/A)	115,952 (N/A)	172,183 (N/A)	120,671 (N/A)	242,661 (N/A)	894,910 (N/A)
**Race/Ethnicity^†^**							
Hispanic/Latino	411,775 (27.4)	200,397 (31.0)	38,553 (34.4)	54,457 (33.0)	38,094 (32.0)	69,293 (27.8)	211,378 (24.6)
White, non-Hispanic	754,801 (50.2)	292,930 (45.4)	42,384 (37.8)	68,887 (41.8)	53,772 (45.1)	127,887 (51.3)	461,871 (53.8)
Black, non-Hispanic	176,059 (11.7)	79,291 (12.3)	16,355 (14.6)	21,308 (12.9)	14,228 (11.9)	27,400 (11.0)	96,768 (11.3)
Asian/Pacific Islander, non-Hispanic	50,224 (3.3)	21,243 (3.3)	4,716 (4.2)	6,109 (3.7)	3,556 (3.0)	6,862 (2.8)	28,981 (3.4)
American Indian/Alaska Native, non-Hispanic	23,396 (1.6)	12,887 (2.0)	2,249 (2.0)	3,653 (2.2)	2,610 (2.2)	4,375 (1.8)	10,509 (1.2)
Multiracial/Other race	87,910 (5.8)	38,923 (6.0)	7,860 (7.0)	10,490 (6.4)	6,911 (5.8)	13,662 (5.5)	48,987 (5.7)
Missing/Unknown*	1,367,663 (N/A)	576,352 (N/A)	100,762 (N/A)	149,009 (N/A)	108,067 (N/A)	218,514 (N/A)	791,311 (N/A)
**Underlying condition** ^§^							
Any	114,934 (30.3)	43,388 (27.6)	6,334 (23.7)	10,203 (26.4)	8,206 (28.8)	18,645 (29.5)	71,546 (32.2)
None	264,313 (69.7)	113,621 (72.4)	20,426 (76.3)	28,386 (73.6)	20,280 (71.2)	44,529 (70.5)	150,692 (67.8)
Missing/Unknown*	2,492,581 (N/A)	1,065,014 (N/A)	186,119 (N/A)	275,324 (N/A)	198,752 (N/A)	404,819 (N/A)	1,427,567 (N/A)
**Known condition^¶^**	421,078 (14.7)	176,766 (14.5)	30,665 (14.4)	43,765 (13.9)	32,122 (14.1)	70,214 (15.0)	244,312 (14.8)
Chronic lung disease	26,937 (6.4)	10,521 (6)	786 (2.6)	2,495 (5.7)	2,316 (7.2)	4,924 (7.0)	16,416 (6.7)
Disability**	4,162 (1.0)	1,992 (1.1)	243 (0.8)	497 (1.1)	411 (1.3)	841 (1.2)	2,170 (0.9)
Immunosuppression	3,495 (0.8)	1,373 (0.8)	196 (0.6)	323 (0.7)	237 (0.7)	617 (0.9)	2,122 (0.9)
Diabetes mellitus	4,030 (1.0)	1,104 (0.6)	63 (0.2)	133 (0.3)	237 (0.7)	671 (1.0)	2,926 (1.2)
Psychological	3,055 (0.7)	1,176 (0.7)	23 (0.1)	153 (0.3)	231 (0.7)	769 (1.1)	1,879 (0.8)
Cardiovascular disease	3,103 (0.7)	1,133 (0.6)	266 (0.9)	239 (0.5)	163 (0.5)	465 (0.7)	1,970 (0.8)
Current/Former smoker	15,362 (3.6)	798 (0.5)	37 (0.1)	42 (0.1)	39 (0.1)	680 (1.0)	14,564 (6.0)
Severe obesity^††^	1,865 (0.4)	566 (0.3)	32 (0.1)	109 (0.2)	121 (0.4)	304 (0.4)	1,299 (0.5)
Chronic kidney disease	796 (0.2)	336 (0.2)	80 (0.3)	77 (0.2)	44 (0.1)	135 (0.2)	460 (0.2)
Hypertension	1,788 (0.4)	272 (0.2)	43 (0.1)	20 (0)	29 (0.1)	180 (0.3)	1,516 (0.6)
Autoimmune disease	919 (0.2)	305 (0.2)	17 (0.1)	45 (0.1)	56 (0.2)	187 (0.3)	614 (0.3)
Chronic liver disease	407 (0.1)	137 (0.1)	22 (0.1)	24 (0.1)	22 (0.1)	69 (0.1)	270 (0.1)
Substance abuse/use	355 (0.1)	72 (<0.1)	1 (<0.1)	1 (<0.1)	6 (<0.1)	64 (0.1)	283 (0.1)
Other	10,100 (2.4)	3,511 (2.0)	665 (2.2)	725 (1.7)	581 (1.8)	1,540 (2.2)	6,589 (2.7)
**Outcome**							
**Hospitalized**							
Yes	30,229 (2.5)	11,882 (2.3)	4,294 (4.6)	1,983 (1.5)	1,598 (1.6)	4,007 (2.0)	18,347 (2.7)
No	1,172,310 (97.5)	514,834 (97.7)	88,786 (95.4)	132,108 (98.5)	96,021 (98.4)	197,919 (98.0)	657,476 (97.3)
Missing/Unknown*	1,669,289 (N/A)	695,307 (N/A)	119,799 (N/A)	179,822 (N/A)	129,619 (N/A)	266,067 (N/A)	973,982 (N/A)
**ICU admission**							
Yes	1,973 (0.8)	866 (0.8)	288 (1.8)	168 (0.6)	131 (0.6)	279 (0.6)	1,107 (0.8)
No	252,961 (99.2)	109,234 (99.2)	16,091 (98.2)	25,968 (99.4)	20,574 (99.4)	46,601 (99.4)	143,727 (99.2)
Missing/Unknown*	2,616,894 (N/A)	1,111,923 (N/A)	196,500 (N/A)	287,777 (N/A)	206,533 (N/A)	421,113 (N/A)	1,504,971 (N/A)
**Died**							
Yes	654 (<0.1)	178 (<0.1)	52 (<0.1)	30 (<0.1)	27 (<0.1)	69 (<0.1)	476 (0.1)
No	1,409,626 (100)	620,989 (100)	111,437 (100)	162,971 (100)	115,664 (100)	230,917 (100)	788,637 (99.9)
Missing/Unknown*	1,461,548 (N/A)	600,856 (N/A)	101,390 (N/A)	150,912 (N/A)	111,547 (N/A)	237,007 (N/A)	860,692 (N/A)

**Abbreviations:** ICU = intensive care unit; N/A = not available.

* Data are missing for more than 47% of cases. Percentages are calculated from among those with available information only.

^†^ Cases reported as Hispanic or Latino were categorized as “Hispanic/Latino” regardless of availability of race data.

^§^ Underlying conditions were defined based on the categories included in the COVID-19 Case Report Form including diabetes mellitus, hypertension, severe obesity, cardiovascular disease, chronic renal disease, chronic liver disease, chronic lung disease (asthma, emphysema, and chronic obstructive pulmonary disease [COPD]), other (specified) chronic diseases, other (specified) underlying condition or risk behavior, immunosuppressive conditions, autoimmune conditions, being a current or former smoker, substance abuse or misuse, disability, and psychological/psychiatric condition. Although obesity in children is defined using body mass index percentile, these data are drawn from the COVID-19 Case Report Form, in which severe obesity is defined as noted.

^¶^ Status of underlying health conditions were known for 421,078 persons aged 0–24 years. Condition status was classified as “known” if any of the conditions included in the COVID-19 Case Report Form were reported as present or absent. Proportion of cases with each individual condition were calculated among persons with known condition status.

** Disability included neurologic or neurodevelopmental disorders, intellectual or physical disability, and vision or hearing impairment.

^††^ Body mass index ≥40 kg/m^2^. Although obesity in children is defined using body mass index percentile, these data are drawn from the COVID-19 Case Report Form, in which severe obesity is defined as noted.

Among persons aged 0–24 years, weekly incidence was higher in each successively increasing age group; weekly incidence among adults aged 25–64 years and ≥65 years exceeded that among children and adolescents aged 0–13 years throughout the review period (Figure 1). Weekly incidence was highest during the final week of the review period (the week of December 6) in all age groups: 99.9 per 100,000 (0–4 years), 131.4 (5–10 years), 180.6 (11–13 years), 255.6 (14–17 years), and 379.3 (18–24 years). Trends in weekly incidence for all age groups aged 0–17 years paralleled those observed among adults since June. The trend in incidence among young adults aged 18–24 years had a distinct and more prominent peak during the week of September 6.

**FIGURE 1:**
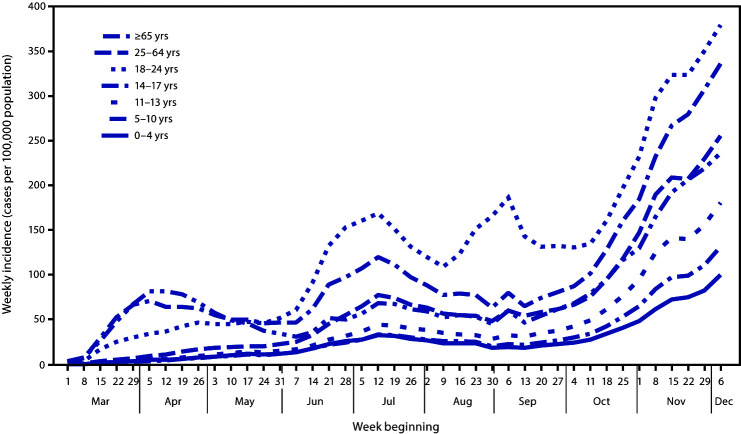
COVID-19 weekly incidence,*^,^† by age group — United States, March 1–December 12, 2020^§^ **Abbreviation:** COVID-19 = coronavirus disease 2019. * The 7-day moving average of new cases (current day + 6 preceding days/7) was calculated to smooth expected variation in daily case counts. ^†^ Incidence was calculated per 100,000 population using 2019 U.S. Census population estimates obtained from Kids Count Data Center (https://datacenter.kidscount.org/data). ^§^ Data included through December 12, 2020, so that each week has a full 7 days of data.

Weekly SARS-CoV-2 laboratory testing among children, adolescents, and young adults increased 423.3% from 435,434 tests during the week beginning May 31 to 2,278,688 tests during the week beginning December 6 (Figure 2).^¶¶^ At their peak during the week of November 15, tests conducted among children and adolescents aged 0–17 years represented 9.5% of all tests performed, and tests among young adults aged 18–24 years represented 15.3% (Supplementary Figure 1, URL https://stacks.cdc.gov/view/cdc/100246). As observed in trends in incidence, weekly percentage of positive test results among children and adolescents paralleled those of adults, declining between July and September, and then increasing through December (Supplementary Figure 2, URL https://stacks.cdc.gov/view/cdc/100246). Percentage of positive test results among young adults aged 18–24 years peaked earlier in June and increased slightly in late August; this was not observed among other age groups. In contrast to incidence, percentage of positive test results among children and adolescents aged 11–17 years exceeded that among younger children for all weeks and that of all age groups since the week beginning September 6; test volumes over time were lowest among children and adolescents aged 11–13 years, suggesting incidence among these age groups might be underestimated.

**FIGURE 2:**
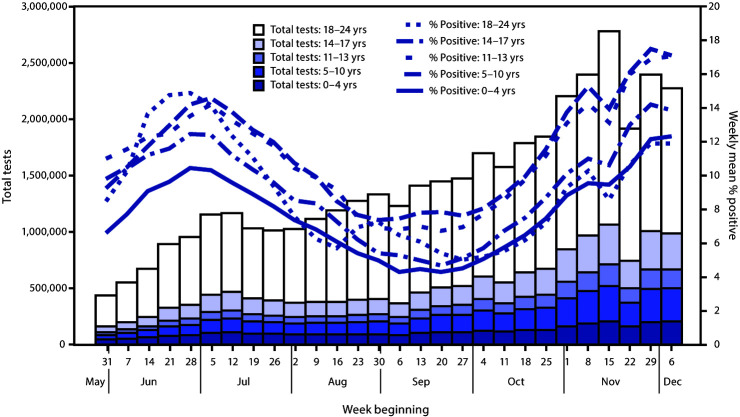
Weekly test volume and percentage of SARS-CoV-2-positive test results* among persons aged 0–24 years, by age group — United States, May 31–December 12, 2020^†^ * By reverse transcription–polymerase chain reaction testing. ^†^ Data included through December 12, 2020, so that each week has a full 7 days of data.

Among cases reviewed, data were available for 41.9%, 8.9%, and 49.1% of cases for hospitalizations, intensive care unit (ICU) admissions, and deaths, respectively. Among children, adolescents, and young adults with available data for these outcomes, 30,229 (2.5%) were hospitalized, 1,973 (0.8%) required ICU admission, and 654 (<0.1%) died (Table), compared with 16.6%, 8.6%, and 5.0% among adults aged ≥25 years, respectively. Among children, adolescents, and young adults, the largest percentage of hospitalizations (4.6%) and ICU admissions (1.8%) occurred among children aged 0–4 years. Among 379,247 (13.2%) children, adolescents, and young adults with COVID-19 and available data on underlying conditions, at least one underlying condition or underlying health condition was reported for 114,934 (30.3%), compared with 836,774 (60.4%) among adults aged ≥25 years.

## Discussion

Reported weekly incidence of COVID-19 and percentage of positive test results among children, adolescents, and young adults increased during the review period, with spikes in early summer, followed by a decline and then steeply increased in October through December. In general, trends in incidence and percentage of positive test results among preschool-aged children (0–4 years) and school-aged children and adolescents (5–17 years) paralleled those among adults throughout the summer and fall, including during the months that some schools were reopening or open for in-person education. In addition, reported incidence among children, adolescents, and young adults increased with age; among children aged 0–10 years, incidence and percentage of positive test results were consistently lower than they were among older age groups. Case data do not indicate that increases in incidence or percentage of positive test results among adults were preceded by increases among preschool- and school-aged children and adolescents. In contrast, incidence among young adults (aged 18–24 years) was higher than that in other age groups throughout the summer and fall, with peaks in mid-July and early September that preceded increases among other age groups, suggesting that young adults might contribute more to community transmission than do younger children.

Findings from national case and laboratory surveillance data complement available evidence regarding risk for transmission in school settings. As of December 7, nearly two thirds (62.0%) of U.S. kindergarten through grade 12 (K–12) school districts offered either full or partial (hybrid with virtual) in-person learning.*** Despite this level of in-person learning, reports to CDC of outbreaks within K–12 schools have been limited,^†††^ and as of the week beginning December 6, aggregate COVID-19 incidence among the general population in counties where K–12 schools offer in-person education (401.2 per 100,000) was similar to that in counties offering only virtual/online education (418.2 per 100,000).^§§§^ Several U.S. school districts with routine surveillance of in-school cases report lower incidence among students than in the surrounding communities^¶¶¶^ (*2*), and a recent study found no increase in COVID-19 hospitalization rates associated with in-person education (*3*). In contrast to the evidence regarding K–12 school reopenings, previous studies provide evidence for increased community incidence in counties where institutions of higher education reopened for in-person instruction (*4*), and presented case surveillance data showed unique trends.

Success in preventing introduction and transmission of SARS-CoV-2 in schools depends upon both adherence to mitigation strategies in schools and controlling transmission in communities (*5*). In settings with low community incidence, where testing and effective mitigation strategies were in place, studies of in-school transmission have provided preliminary evidence of success in controlling secondary transmission in child care centers and schools (*6*–*8*). Schools provide a structured environment that can support adherence to critical mitigation measures to help prevent and slow the spread of COVID-19. When community transmission is high, cases in schools should be expected, and as with any group setting, schools can contribute to COVID-19 transmission (*5*–*7*), especially when mitigation measures, such as universal and proper masking, are not implemented or followed.

The findings in this report are subject to at least four limitations. First, COVID-19 incidence is likely underestimated among children and adolescents because testing volume among these age groups was lower than that for adults, the rate of positive test results was generally higher among children and adolescents (particularly those aged 11–17 years) than that among adults, and testing frequently prioritized persons with symptoms; asymptomatic infection in children and adolescents occurs frequently (*9*). Second, data on race/ethnicity, symptom status, underlying conditions, and outcomes are incomplete, and completeness varied by jurisdiction; therefore, results for these variables might be subject to reporting biases and should be interpreted with caution. Future reporting would be enhanced by prioritizing completeness of these indicators for all case surveillance efforts. Third, the reporting of laboratory data differs by jurisdiction and might underrepresent the actual volume of laboratory tests performed; as well, reporting of laboratory and case data are not uniform.**** Finally, the presented analysis explores case surveillance data for children, adolescents, and young adults; trends in cases among teachers and school staff members are not available because cases are not routinely reported nationally by occupations other than health care workers.

Lower incidence among younger children and evidence from available studies (*2*–*8*) suggest that the risk for COVID-19 introduction and transmission among children associated with reopening child care centers and elementary schools might be lower than that for reopening high schools and institutions of higher education. However, for schools to operate safely to accommodate in-person learning, communities should fully implement and strictly adhere to multiple mitigation strategies, especially universal and proper masking, to reduce COVID-19 incidence within the community as well as within schools to protect students, teachers, and staff members. CDC recommends that K–12 schools be the last settings to close after all other mitigation measures have been employed and the first to reopen when they can do so safely (*10*). CDC offers tools^††††^ to help child care programs, schools, colleges and universities, parents, and caregivers plan, prepare, and respond to COVID-19, thereby helping to protect students, teachers, and staff members and slowing community spread of COVID-19.

**Summary**

**What is already known about this topic?**

Studies have consistently shown that children, adolescents, and young adults are susceptible to SARS-CoV-2 infections. Children and adolescents have had lower incidence and fewer severe COVID-19 outcomes than adults.

**What is added by this report?**

COVID-19 cases in children, adolescents, and young adults have increased since summer 2020, with weekly incidence higher in each successively increasing age group. Trends among children and adolescents aged 0–17 years paralleled those among adults.

**What are the implications for public health practice?**

To enable safer in-person learning, schools and communities should fully implement and strictly adhere to multiple mitigation strategies, especially universal and proper mask wearing, to reduce both school and community COVID-19 incidence to help protect students, teachers, and staff members from COVID-19.

## Footnotes

* CDC official counts of COVID-19 cases and deaths, released daily at https://www.cdc.gov/coronavirus/2019-ncov/cases-updates/cases-in-us.html, are aggregate counts from reporting jurisdictions. Individual-level case report data were available for approximately 75% of the aggregate number of confirmed cases. Cases reported without sex or age data and in persons repatriated to the United States from Wuhan, China, or the Diamond Princess cruise ship were excluded from this analysis.

† Underlying health conditions were defined based on the categories included in the COVID-19 Case Report Form. https://www.cdc.gov/coronavirus/2019-ncov/downloads/pui-form.pdf.

§ Weekly incidence date based on the earliest symptom onset date reported for each COVID-19 case. If symptom onset date was missing, earliest onset date was populated with the earliest date in a series of variables submitted by the jurisdiction, including symptom resolution date, positive specimen date, diagnosis date, specimen collection date (for sputum, nasopharyngeal, oropharyngeal, or other specimen type), hospital or ICU admission or discharge date, date of death, or the date of case reporting to CDC.

¶ Population estimates used in calculating incidence were obtained from the Kids Count Data Center. https://datacenter.kidscount.org/data.

** COVID-19 Electronic Laboratory Reporting data submitted by state health departments from all laboratories performing SARS-CoV-2 RT-PCR testing were used for 44 states, the District of Columbia, Guam, Marshall Islands, and Northern Mariana Islands. SARS-CoV-2 RT-PCR testing data from a subset of public health, commercial, and reference laboratories were used for six states for which data were not directly submitted by state health departments (Maine, Missouri, Ohio, Oklahoma, Washington, and Wyoming), Puerto Rico, and the U.S. Virgin Islands. The data might not include results from all testing sites within a jurisdiction and therefore might reflect the majority of, but not all, SARS-CoV-2 RT-PCR tests in the United States. The data represent laboratory test totals, not individual persons tested, and exclude antibody and antigen tests.

†† 45 C.F.R. part 46, 21 C.F.R. part 56; 42 U.S.C. Sect. 241(d); 5 U.S.C. Sect. 552a; 44 U.S.C. Sect. 3501 et seq.

§§ In 2019, children and adolescents of Hispanic/Latino ethnicity accounted for 26% of children aged 0–17 years; children and adolescents of non-Hispanic Black race accounted for 14% of children aged 0–17 years; and children and adolescents of non-Hispanic White race accounted for 50% of children and adolescents aged 0–17 years in the United States. https://datacenter.kidscount.org/data/customreports/1/103/compared,single#ind103.

¶¶ The percentage increase in test volume between the weeks beginning May 31 and December 6 by age group were 328.0% (0–4 years), 644.1% (5–10 years), 669.2% (11–13 years), 536.3% (14–17 years), and 368.1% (18–24 years).

*** https://www.mchdata.com/covid19/schoolclosings.

††† In addition to routine case surveillance reports, CDC receives regular updates from state, local, and tribal health departments, as well as various school districts. School-based outbreaks have been periodically reported to CDC at the time jurisdictions request technical assistance. In the context of childcare and K–12 schools, requests for assistance have more frequently been in response to a single case or small clusters of cases. Reports of large outbreaks in these settings have been rare.^ §§§^ Data presented are for the week beginning December 6, 2020. Aggregate case incidence is the rate derived after summing the case and population values for counties that currently have that K–12 teaching plans. Among the 2,717 counties having school districts with currently known teaching plans, 1,696 had school districts with differing methods. For these counties, case incidence and positive test result data are proportionately allocated into a specific plan based on the ratio of total enrollment for school districts that currently have that plan type to the total enrollment for all school districts in that county. Population estimates were obtained from the Vintage 2019 Bridged-Race Postcensal Population Estimates for Calculating Vital Rates (https://www.cdc.gov/nchs/nvss/bridged_race/data_documentation.htm). County-level case counts were obtained from CDC County Aggregate figures (extracted December 28, 2020). School enrollment data and school reopening plans were obtained from MCH Strategic Data (https://www.mchdata.com/covid19/schoolclosings, extracted December 28, 2020). Data were extracted and analyzed by the Johns Hopkins University Applied Physics Laboratory.

¶¶¶ Cases and enrollment reported by the New York Department of Health (https://schoolcovidreportcard.health.ny.gov/#/summary)since October 12, 2020, are analyzed on the National COVID-19 School Response Dashboard (https://covidschooldashboard.com) which presents case and infection rates.

**** Percentage positive for laboratory data for some states relied on data reported directly to CDC from public health laboratories and a sample of six large commercial laboratories.

†††† https://www.cdc.gov/coronavirus/2019-ncov/community/schools-childcare/index.html.
